# Implications of COVID-19 for resumption of sport in South Africa: A South African Sports Medicine Association (SASMA) position statement – Part 1

**DOI:** 10.17159/2078-516X/2020/v32i1a8454

**Published:** 2020-01-01

**Authors:** DA Ramagole, DC Janse van Rensburg, L Pillay, P Viviers, P Zondi, J Patricios

**Affiliations:** 1 Section Sports Medicine & Sport, Exercise Medicine and Lifestyle Institute (SEMLI), Faculty of Health Sciences, University of Pretoria, Pretoria, South Africa; 2 Medical Board Member, International Netball Federation, Manchester, UK; 3 Campus Health Service, Stellenbosch University, South Africa; 4 Institute of Sport and Exercise Medicine, Division of Orthopaedic Surgery, Department of Surgical Sciences, Faculty of Medicine and Health Sciences, Stellenbosch University, South Africa; 5 FIFA Medical Centre of Excellence, South Africa; 6 Sports Science Institute of South Africa, Newlands, South Africa; 7 South African Sports Confederation and Olympic Committee, Medical Advisory Committee; 8 Wits Institute for Sport and Health (WISH), Faculty of Health Sciences, University of the Witwatersrand, Johannesburg, South Africa

**Keywords:** return to play, coronavirus, pandemic

## Abstract

The significant impact of the coronavirus disease 2019 (COVID-19) pandemic has extended to sport with the cessation of nearly all professional and non-professional events globally. Recreational parks and fitness centres have also closed. A challenge remains to get athletes back to participation in the safest way, balancing the protection of their health while curbing the societal transmission of the virus.

With this Position Statement, the South African Sports Medicine Association (SASMA) aims to guide return-to-sport as safely as possible, in an evidence-based manner, given that COVID-19 is a new illness and new information from experts in various fields continues to emerge. Clinical considerations are briefly described, focusing on a return-to-sport strategy, including education, preparation of the environment, risk stratification of sports and participants, and the practical implementation of these guidelines. The management of the potentially exposed or infected athlete is further highlighted.

It is important that persons charged with managing athletes’ return-to-sport in any environment must be up-to-date with local and international trends, transmission rates, regulations and sport-specific rule changes that might develop as sport resumes. Additionally, such information should be applied in a sports-specific manner, considering individual athlete’s and team needs and be consistent with national legislation.

South Africa, like many other countries, is grappling with the various implications of the coronavirus disease 2019 (COVID-19) pandemic. The pandemic has had a significant effect on individuals, businesses, and society at large. Sport was not spared from the impact with the cessation of all professional and non-professional sport. All recreational parks and fitness centres were closed. South Africans participating in many codes of amateur and elite sport had scheduled events cancelled or postponed and their ability to train was limited.

Government imposed a lockdown period to control and slow down the spread of infection, strongly advocating for preventative measures, including physical distancing and hand sanitising, emphasising that no specific treatment or vaccine is available. The initial ‘hard’ level 5 lockdown was supposed to be for three weeks, but was subsequently increased by two more weeks to further contain the spread of the virus and to give the relevant authorities time to coordinate a national and subnational response. A risk-adjusted approach was thereafter adopted, implementing various levels of societal reintegration to progressively return the country to normality while trying to mitigate the spread of disease.

Carmody et al., describe similar periods of lockdown and what these measures aim to achieve when facing a novel pathogen and pandemic for which most were unprepared.^[1]^ The South African Sports Medicine Association (SASMA) has written a position statement on guidelines for the safe return to sports for athletes, based on literature from experts in various fields. This is intended as a guide, and should be used in conjunction with medical practitioners’ clinical evaluation of athletes and local government guidelines.

## Reducing transmission

Globally, governments have restricted activities to minimise human-to-human transmission of the virus. This activity restriction is aimed at buying time and allowing for international collaboration between governments and local authorities while strategising to allocate medical resources to counter the pandemic. In this period three pillars of the strategy have been identified, namely.^[1]^

1. **Testing:** Suspected cases should be tested where possible, but this has to be done where it is clinically appropriate and preventative strategies should be in place.^[1]^
2. **Contact tracing:** Measures should be in place to follow-up on all confirmed cases of COVID-19 and all contacts traced to identify and stop transmission.^[1]^
3. **Treatment:** All cases should be managed effectively, and governments should be equipped to provide adequate numbers of hospital beds, intensive care unit (ICU) capacity, ventilators and healthcare professionals.^[1]^

### Organised events

Five key questions address risk factors when allowing organised events. These are highlighted by the World Health Organisation (WHO) and have been incorporated into a risk assessment tool developed to guide event organisers in mitigating the spread of COVID-19.^[2]^ The questions are as follows:

- Is there documented active local transmission of COVID-19 (community spread) in the country that will be hosting the events?
- Are there multiple venues, cities or countries hosting the event, or is it held in a single venue?
- re the participants and spectators also from other international destinations? Do those countries have documented local transmission of COVID-19 which is still active i.e. documented community spread?
- Are most of the participants or spectators expected to be high-risk for becoming infected and developing severe COVID-19 disease? This category includes people over 65 years of age or people with underlying chronic medical conditions.
- Does the event involve contact or non-contact sports (where contact sports are considered a higher risk for transmission of COVID-19)?

One method of mass gathering risk stratification is colour coding, as proposed by Carmody et al^[1]^ (Table 1).

## Clinical considerations

The clinical presentation of severe acute respiratory syndrome coronavirus 2 (SARS-CoV-2) differs in individuals, ranging from mild to severe.^[3]^

### Cardiorespiratory complications

Respiratory system illnesses are a major characteristic of the disease and in athletes it may result in a significant loss of training time, so requires special mention.^[4]^ There is also an increased risk of cardiac complications in patients with a history of viral infection.^[5]^ Persons with influenza infections have a higher incidence of cardiovascular complications, like myocarditis, heart failure and acute myocardial infarction, in comparison to non-infected individuals.^[6]^ Athletes who have had a febrile viral infection, including SARS-CoV-2, are at risk of developing cardiovascular complications, as shown by elevated troponin levels in affected individuals.^[7]^ Athletes should avoid competitive sports for three to six months if they sustain a myocarditis,^[4]^ and a risk stratified return-to-sport paradigm should be implemented.^[6]^ Guidance on return to sport for these athletes should include a cardiology review supported by an ECG, echocardiogram, and a slow gradual retraining and return to sports programme.^[7]^ This gradual retraining and loading is also necessary for the prevention of injuries and acute illnesses, and to maintain the psychological wellbeing of athletes.^[8]^

### Mental health

This “cool off” period will negatively affect the recreational and professional athlete’s level of conditioning and ability to qualify and compete. Forced training restriction is also associated with alterations in mood and feelings of depression in athletes, and has been described in approximately 50% of athletes during South Africa’s lockdown.^[9]^ Mental fatigue due to the lockdown will thus need to be addressed to help them deal with this pandemic and the consequences of training restrictions.^[10]^

### Exercise and immune function

It has been widely publicised that exercise helps to improve the immune system and that during lockdown exercise should be continued as advised by the WHO, but the greatest responsibility is to limit the exposure to and spread of COVID-19.^[11]^ It is recognised that moderate levels of activity are required to assist in improving immunity, and that people who are physically active are likely to have a less severe form of illness and may recover earlier than those who are less active.^[11]^ Athletes should be cautioned to avoid marked changes in load and high-intensity bouts of exercise as this may reduce immunity. These high-intensity activities are described as ‘activities performed by highly athletic individuals’.^[11]^ In addition, with the approaching winter in South Africa, it is advisable to have a flu vaccine. This does not protect against COVID-19 but may reduce the burden of illness during the vulnerable winter months.^[12]^

### Body composition and diet

Although many athletes may continue to exercise under lockdown (64%),^[9]^ a change in training habits and reduction in training load may have resulted in changes in body composition. There is a concern that athletes will have deconditioned and may succumb to injuries should they return to full activity prematurely or in an uncontrolled manner. Dietary habits and nutrition may also be impacted negatively.^[9]^ The importance of balanced nutrition, good hydration, adequate Vitamin D and continuing to exercise under lockdown has been emphasised.^[10]^

Considering all of the aforementioned factors, a return-to-sports strategy has to be structured safely and plans should include a staged increase in exercise levels.

### Return-to-sport

Several international and South African associations have presented plans on restarting competitive sport post-lockdown. These include specific clinical work-ups for the participating athletes.^[7,13]^ Both the athletes and administrators need to be aware of how to return to sport as safely as possible.

The WHO has published a guideline ‘Mass gathering mitigation checklist for COVID-19: addendum for sporting events’ with a checklist of measures that have been implemented to reduce the risk of transmission.^[2]^ Based on the scoring system of 0 to 6, where 0 is a negligible risk, and 6 is a very high risk, event organisers can then classify the risk level, formulate a risk-appropriate return-to-play strategy, and gauge their preparedness. This complete risk evaluation can be found in the separate supplementary document.

The Australian Institute of Sports released a detailed white paper on exercise loading after reduced exercise which can be used as a guide to protect against injuries. This paper recommends that the length of reduced exercise and the percentage of exercise reduction should be used to determine the period it will need to return to the previous level of conditioning.^[14]^

The South African Sports Confederation and Olympic Committee (SASCOC) released a media statement wherein they stated that they cannot dictate to federations how to phase in their return-to-sport strategies. Instead, they advised that each federation’s medical team, in conjunction with other medical authorities, should make use of all available knowledge and resources to formulate a sport-specific plan on how to safely resume sporting activities.^[15]^

In compliance with the government’s risk-adjusted strategy to reduce coronavirus transmission risk and address the need for a phased return-to-sport, a stepwise return to normality has been recommended.^[1]^ These levels of alert have also been adopted by the Wits Institute of Sport (WISH) on risk assessment and return-to-sport^[16]^ (Table 2).

As the risk-adjusted levels become more lenient, the ideal is to achieve a return-to-sport participation whilst protecting athletes and others from infection. It is important to note that all guidelines should be in tandem with government protocols presented by the National Institute of Communicable Diseases (NICD) and the Department of Health (DOH).

## SASMA guidelines

The South African Sports Medicine Association (SASMA) recommends that, as a minimum, the following be incorporated into any post-COVID-19 return-to-sport strategy:

1. Education
2. Preparing the environment
3. Risk stratifying the sport
4. Risk stratifying the participants
5. Practical implementation of mitigating measures of different sports

1. Education
  - Ensuring that there is continuous education of athletes and staff regarding physical distancing, hand hygiene, respiratory etiquette and mask-wearing.
  - Displaying posters around training areas and change rooms reminding everyone about the aforementioned issues.
  - Forbidding team handshakes or contact celebrations.
  - Appointing a health officer to ensure compliance with all these aspects.
  - Educating athletes regarding temporary rule changes that may be adopted by sporting bodies, both local and international (e.g. no spitting on fields or not using saliva to shine cricket balls).
2. Preparing the environment
  - Ensuring that there is sufficient access to “non-touch” soap dispensing and running water as a minimum. Alternatively, 70% alcohol-based sanitiser must be provided.
  - Cleaning thoroughly and regularly with appropriate products of all contact surfaces (0.5% sodium hypochlorite) before and after sports participation.
  - Ensuring closed areas are ventilated, physical distancing (at least two meters) of an athlete not participating is enforced and face masks are worn (e.g. change rooms during half-time).
  - Washing of kit by the player themselves as per guidelines (in water of at least 60 degrees Celsius where possible).
  - Allocating a dedicated room in case temporary isolation is required.
3. Risk stratifying the sport
  - Stratifying individual sports with no physical contact as low risk (e.g. singles tennis and golf) while classifying contact and collision sports (team or individual, such as football and rugby) as high risk.
  - Considering the number of essential persons required at the event, as well as spectators, media and non-essential staff in the risk strategy.
  - Accounting for the ventilation of the playing area where unventilated areas will be considered as higher risk while those held outdoors will be considered as lower risk.
4. Risk stratifying the participants
  - Daily screening, aligned to the recommendations of the DOH, using an App or paper-based questions to identify symptomatic individuals prior to arriving at training sessions.
  - The taking of temperatures daily when entering the sporting environment.^[2]^
  - Identifying and preventing the attendance and participation of higher-risk participants (those older than 60 years and those with comorbid diseases).
5. Practical implementation of guidelines
  - Initially forbidding the gathering of any groups during events (no parents etc., only officials and competing individuals).
  - In lockdown level 3, Minister Nkosazana Dlamini Zuma indicated that more time will be allowed for exercise as long as it is well organised with the observation of social distancing and healthy practices. Non-contact sports may resume without spectators.^[17]^
  - Recommending outlines on when not to come to training and what the reporting lines are e.g. coach, manager, and health authorities.
  - Avoiding public transport to get to training/matches.
  - Detailing and availing an action plan in the case of a suspicious case (including targeted tracing) – this may need medical guidance (even remotely).
  - Considering the higher risk of injury in the return-to-sport strategy.^[18,19]^

SASMA acknowledges that several federations in South Africa have drafted sports-specific guidelines for the potentially exposed and the infected athlete. Based on international guidelines, SASMA recommends the following approach:

1. All athletes:
  - Ensuring physical, psychological and competitive equity to athletes to allow them time to return to their previous level of fitness before major events are rescheduled.^[20]^
  - Upholding the principles of social distancing and hand hygiene.^[7]^
2. Athletes that were exposed to individuals who were affected by COVID-19:
  - Those at high risk (were closer than one metre to the infected person or in contact for more than 15 min), and medium risk (>one metre or less than 15 min contact),^[21]^ need to follow the guidelines for self-quarantine, namely 14 days whilst monitoring for symptoms of COVID-19.
  - Alerting their medical provider if they have symptoms in order to undergo further investigations, including a polymerase chain reaction (PCR) test.^[22]^
  - If testing is positive, following the “infected person protocol” as dictated by the DOH and NICD.^[23]^
  - If they display no symptoms during these 14 days, they may follow a guided return to sport, ensuring load is started at low intensity and progressed.
3. Athletes who were infected and tested positive:
  - These athletes need to avoid exercise for the recommended seven to ten days after cessation of symptoms (approximately 21 days from onset of symptoms).^[9]^
  - They must follow DOH/NICD isolation protocols.^[23]^
  - They should be monitored daily if they are self-isolating. Any worsening of symptoms should be reported to the dedicated health officer.
  - There is no disease-specific treatment to be prescribed for COVID-19; symptomatic treatment is advised.
4. Following 14 days of isolation or recovering from the disease:
  - It is recommended that these athletes have 2 COVID-19 tests at least 48 hours apart that are negative.
  - In any event, clinical monitoring, inflammatory markers and cardiovascular monitoring should be implemented.^[24]^
  - Ensure cardiac screening, which should include a resting and effort ECG, and cardiac echo.^[25]^ The stress ECG must only be done ≥ seven days after the mandatory 14 days isolation and at least seven to ten days after symptom resolution.
  - A cardiologist should clear the athlete before commencing exercise due to the possibility of developing a viral myocarditis.
5. Positive tests after the mandatory isolation period:
  - The above protocol implies a mandatory 21 day period of stand down before an athlete can resume training after a positive test. This can last up to three months or however long it takes until symptoms resolve and the clearance protocol is complete.
  - It is recommended that in these cases^[26]^, the same protocol must be followed as in point 4.
  - The return-to-sport approach must be more cautious in these cases.^[7,27]^
  - These athletes, upon return to training, must be screened daily as per usual.
  - Any recurrence of symptoms must be addressed accordingly and urgently.
6. Other medical conditions:
  - Because COVID-19 has poorer outcomes in cases with comorbidities, such as diabetes mellitus, cardiac disease, hypertension and cancer^[27,28]^, any such existing conditions must be appropriately treated.

## Conclusion

In conclusion, it is important that persons charged with managing athletes’ return-to-sport in any environment must be up-to-date with local and international trends, transmission rates, regulations and sport-specific rule changes that might develop as sport resumes. Additionally, such information should be applied in a sports-specific manner, considering individual athlete’s and team needs and be consistent with national legislation.

## Supplementary Information



## Figures and Tables

**Table 1:**
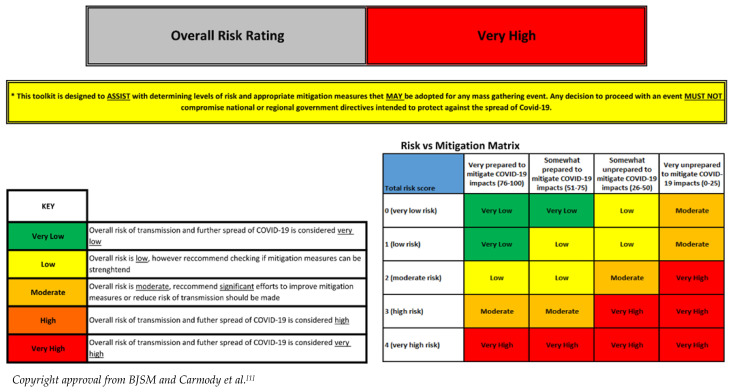
Risk stratification guide

**Table 2:** Alert levels on risk assessment and return-to-sport

Alert level	Environment	Modifications	Examples
5	Exercise in home environment only No use of public gyms	Exercise alone Exercise with asymptomatic family members only	Stretch and strengthen routines • Yoga, Pilates • Home-based online classes • Home treadmill, stationary cycle and rower
4	Exercise in suburbs, open spaces and nearby sports fields	No group exercising • Increase social distancing to at least six metres • Strict hygiene practices (nose-blowing, coughing, spitting) • Exercise alongside each other or staggered instead of behind each other	Jogging, cycling, multiple sprints • Individual sports-specific skills training • Single tennis games
3	3Exercise at training grounds	3Avoid public transport to and from training and wear a cloth mask when travelling • No team travel • Coaching and support staff to wear cloth masks • Disinfecting equipment before and after use • No on-site team or group meetings • Maintain social distancing at six metres minimum for all exercises • Exercise alongside or staggered to each other instead of behind each other • No sharing of water bottles • Shower at home • No spectators at training	3 Sport-specific fitness • Sports-specific skills training with limited equipment e.g. soccer and hockey drills, netball/basketball skills, cricket batting and bowling
32	Exercise at training grounds with full equipment	Train in small groups (maximum five at a time maintaining two metres social distancing during sessions) • No team meetings • Shower at home • No spectators	Non-contact soccer and rugby drills • Hockey, netball and basketball team drills • Squash games
1	Full training and competition	Limit team meetings • No spectators	Matches and games • No spectators

Adapted from and copyright granted by BJSM and Carmody et al.^[1]^
