# Supplementary material for: Implications of COVID-19 for resumption of sport in South Africa: A South African Sports Medicine Association (SASMA) position statement – Part 1
Source: S Afr J Sports Med. 2020 Jan 1;32(1):v32i1a8454. doi: 10.17159/2078-516X/2020/v32i1a8454 (PMC9924566; doi:10.17159/2078-516X/2020/v32i1a8454)
Supplement: Supplementary file 1 [file 2078-516X-32-v32i1a8454-s001.pdf]

Implications of COVID-19 for resumption of sport in South Africa: A South African Sports Medicine Association (SASMA) position statement – Part 1

Guidance for the use of the WHO Mass Gathering Sports Addendum Risk Assessment tools in the context of COVID-19

This provides guidance for organisers of sports events planning mass gatherings during the current outbreak of COVID-19.

Routine planning for mass gatherings includes conducting risk assessments to determine the overall risk of disease spread. In view of the current outbreak of COVID-19, a disease-specific and event-specific risk assessment and mitigation checklist has been developed for use by host countries and organisers of mass gathering, to assess the specific risk of COVID-19.

This tool includes all factors from the general WHO risk assessment and mitigation checklist for mass gatherings, as well as additional factors relating to sporting events, to enable event organisers to determine a more accurate overall risk score.

The following risk assessment and mitigation checklist should be used in conjunction with the WHO Mass Gathering Sports Addendum Guidelines.

In order to accurately provide answers from the following risk assessment and mitigation checklist, organisers must be knowledgeable about the current COVID-19 outbreak. The organisers should reference the daily global COVID-19 situation reports provided by WHO, as well as the national COVID-19 situation reports, if available.

After the tools are completed, the answers need to be entered into the decision matrix for the overall risk score to be determined.

It must be ensured that this risk assessment is conducted with input from local public health authorities and that the necessary personnel with expertise in mass gatherings, risk assessment, epidemiology, and infectious disease control measures are included from the initial stages of planning.

For the overall determination, factors under consideration include:

- the current stage of the COVID-19 outbreak and known transmission dynamics
- the geographical distribution and number of participants, and their individual risk profile
- the risk assessment tool
- the mitigation measures that are currently in place or feasible

Mass gathering risk assessment for COVID-19: Addendum for sporting events

The questions below will enable sport event organisers to review the additional considerations specific to sporting events involving mass gatherings, and thus inform their risk assessment of COVID-19 associated with the event. This will help organisers to understand and manage any additional risk from COVID-19.

The risk assessment should be reviewed and reassessed regularly during the planning phase and updated immediately prior to the transition to the operational phase, especially in light of the rapidly evolving nature of the outbreak. Reference should be made to the latest technical guidance and situation reports on the WHO website.

The risk assessment for COVID-19 associated with the sporting event must be coordinated and integrated with the host country's national risk assessment for COVID-19. As previously mentioned, the person completing the questionnaire should include input from the local public health authorities, consult WHO's latest technical guidance and ensure that there is an up-to-date evaluation of the epidemiological situation.

Risk assessment

Please answer ***Yes (1) or No (0)*** to the following questions to determine a risk assessment score that incorporates factors specific to mass gathering sporting events.

**Additional risk of COVID-19 to the mass gathering sporting event**	**Yes =1**<br>**No =0**
Will the event be held in a country that has documented active local transmission of COVID-19 (community spread)?	
Will the event be held in a single venue or multiple venues/cities/countries?	
Will the event include international participants (athletes and spectators) from countries that have documented active local transmission of COVID-19 (community spread)?	
Will the event include a significant number of participants (athletes or spectators) at higher risk of severe COVID-19 disease (e.g., people over 65 years of age or people with underlying health conditions)?	
Will the event include sports that are considered at higher risk of spread for COVID-19 (e.g. contact sports)?	
Will the event be held indoors?	
**Total COVID-19 risk score**	

Mass gathering mitigation checklist for COVID-19: Addendum for sporting events

Mitigation measures assess the current effort and planning to reduce the risk of spread of COVID-19 disease for the event. As mitigation measures can reduce the overall risk of the mass gathering contributing to the spread of COVID-19, they should be taken into account after the risk assessment has been made to gain a clearer understanding of the overall risk of transmission and further spread of COVID-19, should the mass gathering be held. Together with the risk assessment score, the mitigation measure will contribute to the decision matrix and influence the assessment of the overall risk of transmission and further spread of COVID-19 in relation to the mass gathering.

Topic	Key consideration	Score<br>Yes/Complete =2<br>Maybe/In progress =1<br>No/Not considered =0	Weighting
**Understanding of the overview of the current COVID-19 situation by the event organizers**	Have the relevant organisers and responsible staff been informed about the **latest available guidance on the COVID-19 outbreak** (official web resources available from WHO, CDC, ECDC, UN, local public health authorities)? And are the organizers and staff concerned committed to following the available guidance?		**1**
	Are organisers aware of **global and local daily situation reports** as provided by WHO or local public health authorities?		**1**
	Do the organisers and responsible staff understand the **risks and transmission routes of COVID-19, the steps that event attendees can take to limit spread, the recognized best practices** (including respiratory etiquette, hand hygiene, physical distancing, etc.), and **the travel restrictions** adopted by different countries that may affect the mass gathering?		**1**
**Event emergency preparedness and response plans**	Has a **contingency medical response plan for COVID-19** been developed for this mass gathering sporting event?		**3**
	Does the contingency medical response plan include **information about how attendees should interact with the host country healthcare system** (e.g. hotline/helpline telephone number, medical teams and first-aid points for the mass gathering, local health care system)?		**3**
	Is there an **Emergency COVID-19 Outbreak Response Coordinator/Team** in the organizing committee or other structure for the mass gathering with defined roles and responsibilities, coordinating the health preparedness and response planning for the outbreak?		**2**
	Has the host country or organizer requested **support from WHO and/or local public health authorities**?		**3**
	**Have the organisers of the mass gathering event acquired the following supplies to help reduce the risk of transmission of COVID-19?**		
	Personal protective equipment (e.g. masks, gloves, gowns) for onsite medical personnel		**3**
	Hand sanitiser and alcohol rubs/gels, tissues, frequently replaced soap canisters and closed bins for safe disposal of hygienic materials (e.g. tissues, towels, sanitary products) in washrooms and changing rooms		**3**
	Hand sanitisers and alcohol rubs for all entrances and throughout the venue		**3**
	**If a person feels unwell/ shows symptoms of an acute respiratory infection during the event:**		
	Is there a procedure for athletes or spectators to clearly identify **whom to contact and how to do so** if they or other event participants feel unwell?		**3**
	Is there a protocol on **whom meeting organisers should contact in the host country** to report suspected cases and request testing and epidemiological investigations?		**3**
	Are **first-aid services or other medical services** in place and equipped to support patients with respiratory symptoms?		**2**
	Are there **isolation rooms or mobile isolation units** available onsite?		**2**
	Are there any **designated medical facilities** that manage patients with COVID-19 infection in the host country?		**2**

	Are there **transportation services with trained medical professionals** available to transport critically ill patients with severe acute respiratory infections to a hospital or to evacuate them from the host country, if necessary?		**2**
	Has a **cleaning schedule** been developed to ensure the venue is clean and hygienic – wiping surfaces and any equipment regularly with disinfectant is strongly recommended (before, during and after the event and between each round of competition)?		**3**
	Are there established **screening measures,** including temperature checks in place for participants at the point of entry, venues, routes and on-site medical facilities (first-aid points)? (Please specify in Comments what these screening measures include)		**3**
	Is the host country conducting COVID-19 **laboratory diagnostic tests**? (If Yes, please specify in comments the type of COVID-19 diagnostic test used)		**3**
	Does the host country have a **national public health emergency preparedness and response plan** that can address severe respiratory diseases, including COVID-19?		**2**
	Is there **a preliminary agreement by the host country to provide care** for any COVID-19 cases connected with the mass gathering?		**3**
	If the event is for a duration of 14 days or longer, does the medical response plan for the event include resources and protocols for managing all public health interventions that would be necessary and supporting the national public health authorities if participants are infected and/or become unwell at the event? (If the event is for less than 14 days, please score 0)		**3**
	If the event is for less than 14 days, does the medical response plan include protocols for organisers to notify all participants of possible exposure to COVID-19 if the organisers are made aware of any suspected or confirmed cases that attended the event? (If the event is for 14 days or longer, please score 0)		**3**
**Stakeholder and partner coordination**	Is there an established mechanism for collaboration and coordination between **the health and security sectors**, which is considered as crucial?		**2**
	Are there agreed, clear and easily understood processes in place for **reporting to external multi-sectoral stakeholders** (including surveillance authorities, WHO, CDC, ECDC, etc.) and disseminating risk communication messages (e.g. media)?		**2**
**Command and control**	Is there a decision-making authority/body and an agreed procedure to **modify, restrict, postpone or cancel the mass gathering sporting** event related to the evolving COVID-19 outbreak?		**3**
	Are there arrangements to activate a **strategic health operations centre** if there are suspected COVID-19 cases in connection with the sporting mass gathering?		**2**
	Have the mass gathering organisers and staff **undergone training and exercises** on personal safety procedures and emergency mitigation measures (including those specifically listed in this checklist)?		**3**
**Risk communication**	Is there a **risk communication strategy** for the sporting mass gathering in regard to COVID-19?		**3**
	Is there a **designated person or persons to lead media** activities and tasked with managing all external communications with national and international government officials, the general public, and the media? (If yes, please identify the spokesperson in comments)		**2**
	Has there been **monitoring of national and international media and social media** established for **rumours** to be able to counter them early? (Please explain in the comments what protocols are in place for counter messaging)		**2**
	Has coordination been set up with major **official media channels and social media** sites, such as Twitter, Facebook and Instagram, so that messaging can be coordinated with, and assisted by, the platforms to provide targeted messaging from organisers (including messaging to counter fake news and rumours, and proactive messaging about the status of the mass gathering, including changes)?		**2**

**Public health awareness of COVID-19 before and during the event**	Has **public health advice** on clinical features of COVID-19, preventive measures, especially respiratory etiquette, hand hygiene practices, and physical distancing, been shared with all staff involved in the event, athletes, the public, and personnel of all relevant stakeholders?		3
	Has information on the **at-risk populations** been provided to all athletes, the public and others so that they may make an informed decision on their attendance based on their personal risks?		3
	Has public advice included information on the meaning of the following **measures: quarantine, self-isolation and self-monitoring?**		2
**Surge capacity**	**Are there any surge arrangements in place in the event of a public health emergency during the mass gathering - (i.e. suspected and confirmed cases of COVID-19?**		
	Do these surge arrangements include funding for mitigation measures?		3
	Do these surge arrangements include stockpiles of equipment (e.g. personal protective equipment, etc.)		3
	Do these surge arrangements include training of extra staff?		2
	Do these surge arrangements include volunteers?		2
**Specific mitigation measures**	Will there be **daily health checks** of athletes/competitors?		2
	Will the **athletes be separated from other groups,** such as officials, support staff and spectators, to limit transmission?		2
	Are there measures in place to **limit the sharing of equipment, water bottles, towels,** etc.?		3
	Will athletes be given closed **containers to allow for the safe disposal or storing of all hygienic materials** (e.g. tissues, towels, etc.)?		3
	Will the sporting event have **designated seating** for all spectators?		3
	Does the designated **seating provided allow for physical distancing** between spectators (minimum of one metre)?		2
	Have **pre-travel health checks** been performed on all athletes to ensure underlying comorbidities, medications, allergies, etc. are documented?		2
**Sum of mitigation measures**			
**Total mitigation score (%)**<br>**(Sum of mitigation divided by 220) x 100**			

Mass gathering overall risk score: addendum for sporting events

The decision matrix takes the risk score and the mitigation score to provide a colour determination. This colour determination in the matrix below identifies the total risk of transmission and further spread of COVID-19 in relation to the mass gathering. The 'Colour Determination' key in the matrix describes the total risk for each colour.

**Total COVID-19 risk score (from "Risk Assessment" Tab)**	
**Total mitigation score (from "Mitigation Checklist" Tab)**	

**Mass gathering risk assessment for COVID-19: addendum for sporting events**
The questions below will enable sport event organisers to review the additional considerations specific to sporting events involving mass gatherings, and thus inform their risk assessment of COVID-19 associated with the event. This will help organizers to understand and manage any additional risk from COVID-19.<br>The risk assessment should be reviewed and reassessed regularly during the planning phase and updated immediately prior to the transition to the operational phase, especially in light of the rapidly evolving nature of the outbreak. Reference should be made to the latest technical guidance and situation reports on the WHO website.<br>The risk assessment for COVID-19 associated with the sporting event must be coordinated and integrated with the host country's national risk assessment for COVID-19. The person completing the questionnaire should include input from the local public health authorities, consult WHO's latest technical guidance and ensure that there is an up-to-date evaluation of the epidemiological situation.
**Risk assessment:**

Please answer Yes (1) or No (0) to the following questions to determine a risk assessment score that incorporates factors specific to mass gathering sporting events		
**Additional risk of COVID-19 to the mass gathering sporting event**	**Yes =1 No =0**	**Score**
Will the event be held in a country that has documented active local transmission of COVID-19 (community spread)?		0
Will the event be held in a single venue or multiple venues/cities/countries?		0
Will the event include international participants (athletes and spectators) from countries that have documented active local transmission of COVID-19 (community spread)?		0
Will the event include a significant number of participants (athletes or spectators) at higher risk of severe COVID-19 disease (e.g., people over 65 years of age or people with underlying health conditions)?		0
Will the event include sports that are considered at higher risk of spread for COVID-19 (e.g. contact sports)?		0
Will the event be held indoors?		0
**Total COVID-19 risk score**		**0**

Total Risk Assessment Score	Very Prepared to Mitigate COVID-19 Impacts (76-100)	Somewhat Prepared to Mitigate COVID-19 Impacts (51-75)	Somewhat Unprepared to Mitigate COVID-19 Impacts (26-50)	Very Unprepared to Mitigate COVID-19 Impacts (0-25)
0 - Negligible	Very low	Very low	Very low	Very low
1 - Very Low Risk	Very low	Very low	Low	Low
2 - Low Risk	Low	Low	Low	Moderate
3 - Moderate Risk (low-moderate)	Low	Moderate	Moderate	Moderate
4 - Moderate Risk (high-moderate)	Moderate	Moderate	High	Very High
5 - High Risk	High	High	Very High	Very High
6 - Very High Risk	Very High	Very High	Very High	Very High

KEY FOR COLOUR DETERMINATION OF OVERALL RISK	
VERY LOW	Overall risk of transmission and further spread of COVID-19 in relation to the mass gathering is considered <u>very low</u>.
LOW	Overall risk of transmission and further spread of COVID-19 in relation to the mass gathering is considered <u>low</u>. Recommend checking whether mitigation measures can be strengthened.
MODERATE	Overall risk of transmission and further spread of COVID-19 in relation to the mass gathering is considered <u>moderate</u>. Recommend <u>significant</u> efforts to improve mitigation measures or reduce risk of transmission (decrease risk assessment score).
HIGH	Overall risk of transmission and further spread of COVID-19 in relation to the mass gathering is consdiered <u>high</u>. Recommend <u>significant</u> efforts to improve <u>both</u> mitigation measures and reduce risk of transmission (decrease risk assessment score).
VERY HIGH	Overall risk of transmission and further spread of COVID-19 in relation to the mass gathering is considered <u>very high</u>.
